# SafeMotherMedicine: Aiming to Increase Women’s Empowerment in Use of Medications During Pregnancy and Breastfeeding

**DOI:** 10.1007/s10995-020-02903-9

**Published:** 2020-03-17

**Authors:** Kristine Heitmann, Jan Schjøtt

**Affiliations:** 1 Regional Medicines Information and Pharmacovigilance Centre (RELIS Vest), Department of Medical Biochemistry and Pharmacology, Haukeland University Hospital, 5021 Bergen, Norway; 2 Department of Clinical Science, Faculty of Medicine and Dentistry, University of Bergen, 5021 Bergen, Norway

**Keywords:** Pregnancy, Breastfeeding, Drug information centres, Drug use, Risk assessment

## Abstract

**Purpose:**

Approximately 80% of pregnant women use medications. There is a need for evidence based medicines information that provide realistic risk estimates as pregnant and breastfeeding women tend to overestimate the risk of medications. The purpose of this paper is to describe the development and future perspectives of an innovative medicines information service aiming to increase empowerment among pregnant and breastfeeding women.

**Description:**

SafeMotherMedicine (SMM) (www.tryggmammamedisin.no) is a Norwegian medicines information service for pregnant and breastfeeding women. Established in 2011, the service was initially web-based only, in contrast to most teratology information services that at the time mainly operated using telephone and/or e-mail.

**Assessment:**

During the last eight years, SMM has provided close to 30,000 answers promoting appropriate medication use among pregnant and breastfeeding women. SMM launched a telephone-service in 2016, however, the annual number of questions received through the web-based service continues to increase.

**Conclusion:**

The service seems to have fulfilled a previously unmet need of evidence-based, individually tailored information about medications to pregnant and breastfeeding women in Norway. SMM empowers the women to make informed decisions regarding medication use in pregnancy and breastfeeding, thus contributing to person-centred medicine. The web-based design of the service may represent the pregnant and breastfeeding women’s preferred way of communication.

## Significance

*What is already known on this subject?* Pregnant and breastfeeding women tend to overestimate the risk of use of medications indicating a need for medicines information that provide realistic risk estimates promoting appropriate medication use. *What this study adds?* As the first medicines information service directed to pregnant and breastfeeding women in Norway, SafeMotherMedicine was launched in 2011 as a web-based service only, supplemented with a telephone service in 2016. This commentary describes the development and future perspectives of an innovative medicines information service aiming to increase empowerment among pregnant and breastfeeding women, by adjusting our service to the digital era. We postulate that our description of the 8 years experience of SafeMotherMedicine could be of international interest.

## Purpose

### Use of Medications Among Pregnant and Breastfeeding Women

Approximately 80% of pregnant women use medications (Nordeng et al. 2010; Lupattelli et al. 2014b). Over-The-Counter (OTC) medications and medications for acute/short-term illnesses are used by about two-thirds, and close to one-fifth use medications for chronic conditions (Lupattelli et al. 2014b). For many conditions, the benefit of using medications outweighs the potential risks for the fetus. However, pregnant women tend to overestimate the risk (Nordeng et al. 2010; Widnes and Schjøtt 2017), potentially explaining the low adherence that has been observed among this patient group (Lupattelli et al. 2014a). For some conditions, e.g. epilepsy or severe depression, insufficient treatment or low adherence represents a larger risk for the mother and fetus than the medications that are used to treat it.

A study that investigated pregnant women’s perception of risk and benefits of medications, found that the risks and benefit scores were significantly inversely correlated (Mulder et al. 2017). This implies that clear communication of the benefits of the medications may reduce the women’s risk perception, and increase their empowerment with regard to medications.

Women and health care personnel are also concerned about use of medications during breastfeeding in fear of harming their babies, despite that most medications do not readily cross over to breastmilk in significant amounts (Jahnsen et al. 2018; Hale 2019). As there are several health benefits of breastfeeding both for the infant and the mother, the national health authorities guidelines promote exclusive breastfeeding until the infant is 6 months old, followed by continued breastfeeding while gradually introducing solid foods for at least until the infant is 12 months old (The Norwegian Directorate of Health 2017).

### Information Need

Conducting clinical studies with pregnant or breastfeeding women is ethically challenging. This infer that little information is available at the time of market approval of new medications besides from what is known from preclinical and animal studies. When the medication has been on the market for some time, more experience is gained. However, this experience is mostly based on observational data.

The information about medications during pregnancy and breastfeeding in package leaflets and the Norwegian drug formula (Felleskatalogen) is in general restrictive, categorical, potentially outdated or even misleading (Widnes and Schjøtt 2008; Brown et al. 2016). The potential risks of the medications are generally not weighed against the benefit of adequate treatment in these information sources. Reading the package information leaflet may render the women feeling worried about their medication (Widnes et al. 2013), which again may contribute to the low adherence observed in studies (Lupattelli et al. 2014a).

Pregnant women need access to medicines information sources that provide realistic risk estimates to promote appropriate medication use. Thus, many pregnant women have a need for medicines information during pregnancy, with the internet being a widely used information source (Hämeen-Anttila et al. 2013). In addition, the vast majority of the pregnant women use multiple sources when seeking information on medications. Conflicting information has been shown to be common, resulting in anxiety and abstaining from use of the medication (Hämeen-Anttila et al. 2014).

Even though teratology information services was well established in several other countries (Leen-Mitchell et al. 2000; Lim et al. 2009; Schaefer 2011), no medicines information service directed to pregnant and breastfeeding women was available in Norway before 2011. As many pregnant and breastfeeding women use OTC-medications, targeting the women directly would enable them to ask about the use of such agents as well. It was also hypothesised that the establishment of a publicly available service potentially could be timesaving for health care professionals involved in pregnancy care.

The purpose of this paper is to describe a medicines information service that was started as a web-based service only, aiming to increase empowerment among pregnant and breastfeeding women. Future perspectives are also discussed.

## Description

### The Development of SafeMotherMedicine

SafeMotherMedicine (SMM) is run by the Regional Medicines Information and Pharmacovigilance Centre (RELIS), which is a Norwegian network of medicines information centres, localised in all four health regions (Schjøtt 2017). RELIS has been providing information to health care professionals since its establishment in 1995 (Schjøtt 2017). The staff of the centres constitutes of pharmacists and physicians with expertise in searching and critical evaluation of literature as well as communication skills. As 15–20% of the enquiries from health care professionals to RELIS are requests of information on use of medications during pregnancy and lactation (RELIS 2019), the staff at RELIS has acquired extensive experience with searching for and providing teratology information. Importantly, RELIS promote person-centred and personalised medicine with both a holistic and biological perspective in their decision support, which is of relevance with regard to empowerment (Schjøtt 2019).

Based on the extensive experience in RELIS with providing written information to health care professionals, a written web-based design of the service was chosen, in contrast to most teratology information services that at the time mainly operated using telephone and/or e-mail (Leen-Mitchell et al. 2000; Lim et al. 2009; Schaefer 2011). Providing an online service was also in line with the national trend observed among the general public of increased usage of the internet to search for health related information (Statistics Norway 2019a). The internet penetration rate has indeed been shown to be high in Norway. In 2011, more than 90% of the women of fertile age used the internet on a daily basis. In 2018, the usage rates in the same groups was close to 100% (Statistics Norway 2019b). In addition, Norway has recently been reported to be among the top three countries in Europe with regard to the population’s digital skills (Statistics Norway 2017).

**Fig. 1:**
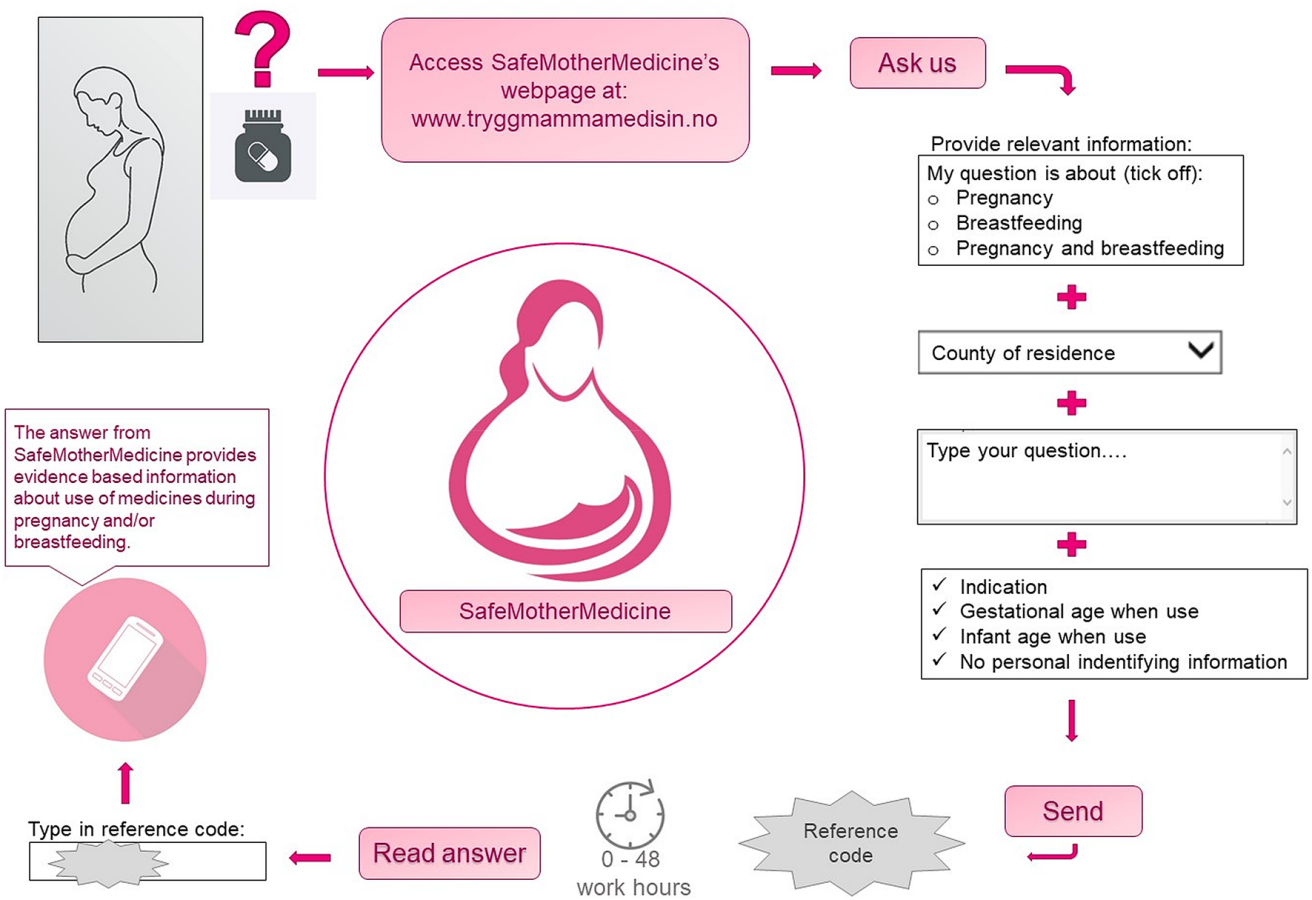
Flow-chart of processing questions to SafeMotherMedicine’s web-based service. RELIS is the Regional Medicines Information and Pharmacovigilance Centres in Norway that serve health care professionals and runs SafeMotherMedicine; a service providing medicines information to pregnant and breastfeeding. Selected graphical elements: Colourbox.com

A web page that enable women to file questions was designed and launched 9th of June 2011, marking the start of the pilot. A button labelled “Ask us” links to the submission page. In addition to the specific question, some additional information is asked for (Fig. 1). A reference code is provided upon submitting. The code is needed to read the answer, which is due within two working days. In addition to the question–answer service, fact sheets with information about use of medications for some common complaints experienced during pregnancy and breastfeeding, such as pain, allergy, heartburn, constipation, nausea, haemorrhoids, and stuffy nose among others, are included on the web page.

All questions are received by one RELIS-centre that is responsible to delete all personal information in the questions, thus securing confidentiality. The questions are divided between the centres based on the women’s county of residence. This sharing of tasks secures alternative staff to answer questions irrespective of illness or acute lack of personnel in one centre. The SMM questions are handled by the same staff at RELIS who answers questions received from healthcare personnel (Schjøtt 2017). This give rise to the opportunity to suggest in the SMM answer that the woman’s physician is welcome to contact RELIS to discuss the case. Furthermore, the staff at SMM is aware of the importance of tailoring the information provided according to whether the question is posed by a pregnant/breastfeeding woman or a health care professional. Previous findings have shown that teratogenic risk perceptions differed between pregnant women and their general practitioners, and may be influenced by the wording in the information presented (Widnes et al. 2013).

All questions received through the web-based service and their corresponding answers are indexed according to the Anatomical Therapeutic Chemical (ATC) classification system for medicines (WHO Collaborating Centre for Drug Statistics Methodology 2019), before stored in full-text in the database which is searchable for staff only. The search can be filtered according to medication and category as indexed by the women (e.g. pregnancy, breastfeeding) (Fig. 1), and the reporting list is sorted by date. This allows for possible reuse of previous answers, increasing the effectiveness of the service. The indexing according to ATC also facilitates the possibility to report summarized statistics useful for planning of medicines information efforts on the SMM website or research efforts. By the end of 2019 the database included more than 28,000 question-and-answer pairs.

### Evidence-Based Information to Promote Empowerment

The main aim to SMM is to promote empowerment among women to make informed decisions regarding own medication use by providing accurate, evidence-based and up-to-date information about use of medications during pregnancy and breastfeeding. The advice and assessments are provided as individually tailored information including realistic risk estimates associated with medication use weighed against the risks of not treating the disease. However, the women are never advised to change the medication without first discussing it with their physician.

A dedicated working group for SMM, consisting of at least one member from each of the four RELIS-centres has been established to ensure that our team are operating in a formalized and coordinated process, and reduce the risk of errors (Schjøtt 2017).

## Assessment

### Achievements

By the end of 2011, close to 1000 questions had been received and answered (RELIS 2019). An evaluation of the pilot was conducted in 2011 with a response rate of 43% (n = 296) (Andresen 2012). In total 96% found the answer from SMM useful. Interestingly, when asked where they first had heard of SMM, 41% reported social media. A qualitative analysis of the comments written in a free-text entry field, revealed useful feedback including a wish of receiving the reference code on SMS and a notification upon ready answer, and having the possibility to pose follow-up questions (Andresen 2012).

**Fig. 2:**
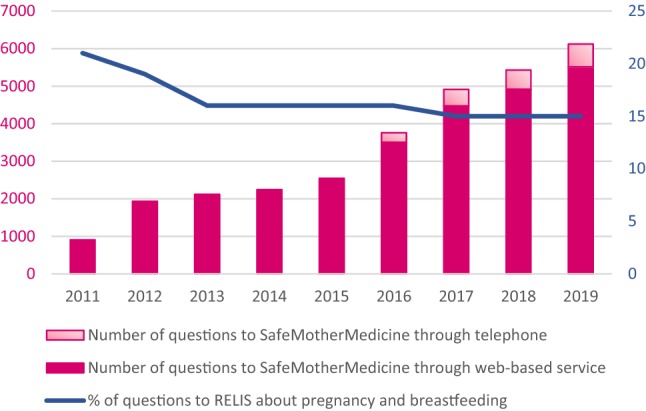
Questions about pregnancy and breastfeeding to RELIS and SafeMotherMedicine 2011–2019. RELIS is the Regional Medicines Information and Pharmacovigilance Centres in Norway that serve health care professionals and runs SafeMotherMedicine; a service providing medicines information to pregnant and breastfeeding

The annual number of questions received through the web-based service continued to increase in the following years, and in 2019 the annual number was over 5500 (Fig. 2). By the end of 2019 SMM has received and answered close to 30,000 questions. In addition, as many as 35,000 unique users of the SMM’s website were registered in 2018 (RELIS 2019). Based on that Norway has approximately 60,000 births per year, this indicates that a large proportion of the pregnant and breastfeeding population uses the service.

The medications most frequently asked about on an annual basis typically include medications for pain, nausea, yeast infection, haemorrhoids, allergy, asthma and mental health (RELIS 2019).

### The Addition of a Telephone Service

As the web-based service does not facilitate follow-up questions for neither the women nor the counsellors, a telephone service was introduced in May 2016 to address this issue. The telephone service enables improved counselling in more complex cases, and is an option for women who prefer oral communication. The user survey for the telephone service (n = 92) revealed a high level of user satisfaction, and 99% of the women stated that they would use the SMM’s telephone service again (Havnen et al. 2018). Furthermore, 74% replied that they would not search for information in other sources after receiving counselling from SMM. From 2017 to 2019 the annual number of questions to the telephone service increased from approximately 450 to 600, which represent less than 10% of the total number of enquiries to SMM (RELIS 2019). This indicates that Norwegian women seem to prefer the web-based service, which partly could be due to not having to relate to specific opening hours, or that the web-based service represents the preferred way of communication. Our experience that the women seem to prefer to communicate in writing over the internet is in accordance with the experience made by the TIS Finland. After the introduction of chat to their service in 2018, a survey showed that the vast majority of the responders preferred chat rather than telephone inspiring to continue the chat service (Malm et al. 2019).

### Future Perspectives

Most pregnant women use the internet to search for information on use of medications during pregnancy (Hämeen-Anttila et al. 2013), including social media and online fora (van Gelder et al. 2019; Palosse-Canaloube et al. 2014), and they use the information in such sources to make decisions about medication use (Lynch et al. 2018). However, assessments of the accuracy of the information provided on social media and online fora about use of medications during pregnancy have shown a high degree of incorrect information (van Gelder et al. 2019; Palosse-Cantaloube et al. 2014). In light of this, offering a web-based service seems prudent in order to meet the women on their preferred communication platform to reach out with accurate information, promoting accurate pharmacotherapy during pregnancy and breastfeeding. However, as the women tend to use multiple information sources, it is of importance to investigate how the information from services such as SMM is weighed against that from other non-evidence based information sources such as social media and online fora. Furthermore, it is of interest to investigate in more detail how the answers from SMM are perceived, interpreted and applied, as well as the women’s perspectives on how best to communicate risk and benefit.

During the 8 years the web-based service has been available, some limitations have been identified that should be addressed in order to improve the service. First of all, 1 of 4 answers are never collected by the women, who may have received information elsewhere or lost their reference number. Second, as of today, it is not possible to notify the women when the answer is ready, which in many cases is before the two working days deadline. Some questions clearly benefit of a quick response, and some questions even loose its relevance if two days pass before the answer is made available or read. The staff at SMM prioritise these kind of questions, and it may be hypothesised that if the women were notified once the answers were ready, the share of non-collected answers could be reduced. Third, it would be more ideal to have a system allowing two-way communication embedded in the web-based service. Opening a chat service in addition to the web-based and telephone service is a possibility. Last, the experience is that the same questions are being asked repeatedly. Publishing more fact sheets about commonly experienced symptoms and a more active fronting of the texts on the web page could be beneficial. Use of a chat-robot to answer FAQs could be considered as a means for increasing the efficiency of SMM.

## Conclusion

In conclusion, the SMM has been a success based on usage rates. The service seems to have fulfilled a previously unmet need of evidence-based, individually tailored information about medications to pregnant and breastfeeding women in Norway. SMM empowers the women to make informed decisions regarding medication use in pregnancy and breastfeeding, thus contributing to person-centred medicine. The web-based design of the service seems to have been right for its time, and may represent the pregnant and breastfeeding women’s preferred way of communication. When further developing the service, efforts have to be made to ensure high quality of all parts of the service, as well as facilitating a sustainable use of resources.
